# Dietary supplementation of bilberry anthocyanin on growth performance, intestinal mucosal barrier and cecal microbes of chickens challenged with *Salmonella *Typhimurium

**DOI:** 10.1186/s40104-022-00799-9

**Published:** 2023-01-20

**Authors:** Sheng Zhang, Yibing Wang, Jinling Ye, Qiuli Fan, Xiajing Lin, Zhongyong Gou, Shouqun Jiang

**Affiliations:** Institute of Animal Science, Guangdong Academy of Agricultural Sciences, State Key Laboratory of Livestock and Poultry Breeding, Key Laboratory of Animal Nutrition and Feed Science in South China, Ministry of Agriculture and Rural Affairs, Guangdong Provincial Key Laboratory of Animal Breeding and Nutrition, Guangzhou, 510640 Guangdong China

**Keywords:** Anthocyanin, Cecal microbe, Chicken, Intestinal mucosal barrier, *Salmonella* Typhimurium

## Abstract

**Background:**

Anthocyanins (AC) showed positive effects on improving the intestinal health and alleviating intestinal pathogen infections, therefore, an experiment was conducted to explore the protective effects of supplemented AC on *Salmonella*-infected chickens.

**Methods:**

A total of 240 hatchling chickens were randomly allocated to 4 treatments, each with 6 replicates. Birds were fed a basal diet supplemented with 0 (CON, and ST), 100 (ACL) and 400 (ACH) mg/kg of AC for d 60, and orally challenged with PBS (CON) or 10^9^ CFU/bird (ST, ACL, ACH) *Salmonella *Typhimurium at d 14 and 16.

**Results:**

(1) Compared with birds in ST, AC supplementation increased the body weight (BW) at d 18 and the average daily gain (ADG) from d 1 to 18 of the *Salmonella*-infected chickens (*P* < 0.05); (2) AC decreased the number of *Salmonella* cells in the liver and spleen, the contents of NO in plasma and inflammatory cytokines in ileal mucosa of *Salmonella*-infected chickens (*P* < 0.05); (3) *Salmonella* infection decreased the ileal villi height, villi height to crypt depth (V/C), and the expression of zonulaoccludins-1 (*ZO*-1), claudin-1, occludin, and mucin 2 (*MUC*2) in ileal mucosa. AC supplementation relieved these adverse effects, and decreased ileal crypt depth (*P* < 0.05); (4) In cecal microbiota of *Salmonella*-infected chickens, AC increased (*P* < 0.05) the alpha-diversity (Chao1, Pd, Shannon and Sobs indexes) and the relative abundance of Firmicutes, and decreased (*P* < 0.05) the relative abundance of Proteobacteria and Bacteroidota and the enrichment of drug antimicrobial resistance, infectious bacterial disease, and immune disease pathways.

**Conclusions:**

Dietary AC protected chicken against *Salmonella* infection via inhibiting the *Salmonella* colonization in liver and spleen, suppressing secretion of inflammatory cytokines, up-regulating the expression of ileal barrier-related genes, and ameliorating the composition and function of cecal microbes. Under conditions here used, 100 mg/kg bilberry anthocyanin was recommended.

## Background

*Salmonella *Typhimurium has strong pathogenicity and causes human and animal infections worldwide. *Salmonella* infection damages the intestinal mucosal barrier of chicken, causing systemic inflammation and immune system disorders, leading to slow growth, diarrhea and even death [1, 2], thus causes serious economic loss in the animal industries. Additionally, infected chickens transmit *Salmonella* to humans through contaminated meat, eggs, or other products, causing public health and safety problems [3]. In production, repeated usage of antibiotics has been reported to cause chronic toxicity in animals, destroying the normal gut microbes, and triggering intestinal inflammation [4]. Many countries and regions have banned or restricted the addition of antibiotics in feed. Thus, under “antibiotic-free” production systems the development of a novel nutritional strategy provides a safer way to prevent *Salmonella* infection in chickens.

Anthocyanins (AC) are widely found in natural berries, fruits, and vegetables, also show positive effects on animal health mainly by anti-inflammatory [5], antioxidant [6], and anti-microbial [7] activities. These properties seem to indicate AC as an effective dietary supplement for alleviating intestinal pathogen infections and improving the intestinal health. Previous studies showed that AC in vitro inhibited the activity of pathogenic bacteria, including *Salmonella *Typhimurium, *Escherichia coli* and *Staphylococcus aureus* [8, 9]. Furthermore, AC improved intestinal health partly through the reduction of barrier permeability and secretion of inflammatory cytokines in mice [10, 11], and by activating the antioxidant adaptive response of intestinal epithelial cells to prevent intestinal inflammation [12, 13]. Our previous study found that supplementation with bilberry AC promoted immune status and suppressed oxidative stress of yellow-feathered chickens [14]. However, alleviation of *Salmonella* infection in yellow-feathered chickens by dint of the protective effect of AC has not been studied yet.

Therefore, the purpose of this study was to explore the effects of supplementation with bilberry AC on the growth performance, intestinal mucosal barrier and composition of cecal microbes in chickens challenged with *Salmonella*, in order to provide an explanation for a potential mechanism, thus to promote the application of AC in feed industry and broiler farming.

## Material and methods

### Animals, diets, and experimental design

Two hundred and forty 1-day-old Lingnan yellow-feathered chickens (male, slow-growing) were randomly allocated into 4 groups with 6 replicates and 10 birds per replicate. The basal diets were formulated in accordance with the recommendation of Nutrient Requirements of Yellow Chickens (Ministry of Agriculture, PRC, 2020) (Table 1) [15].

**Table 1:** Ingredients and nutrient levels of the basal diets

Item	Day 1 to 28	Day 29 to 60
Ingredient, %		
Corn	59.20	67.75
Corn gluten	3.00	1.30
Soybean meal	31.00	25.14
Soybean oil	2.30	2.00
*DL*-Methionine	0.10	0.11
*L*-Lysine HCl	0.20	0.10
Limestone	0.85	1.05
Monocalcium phosphate	1.85	1.05
Zeolite powder	0.20	0.20
NaCl	0.30	0.30
Premix^a^	1.00	1.00
Nutrient levels^b^, % or indicated units		
Metabolic energy, MJ/kg	11.91	12.12
Crude protein	21.00	17.50
Lysine	1.16	0.93
Methionine	0.43	0.39
Methionine + cystine	0.78	0.69
Calcium	0.85	0.72
Total phosphorus	0.69	0.53
Non-phytate phosphorus	0.40	0.26

^a^Premix provided the per kilogram of diets during 1 to 28, and 29 to 60 days of age as reference to previous research [14]

^b^The nutrient levels were calculated values

**Fig. 1:**
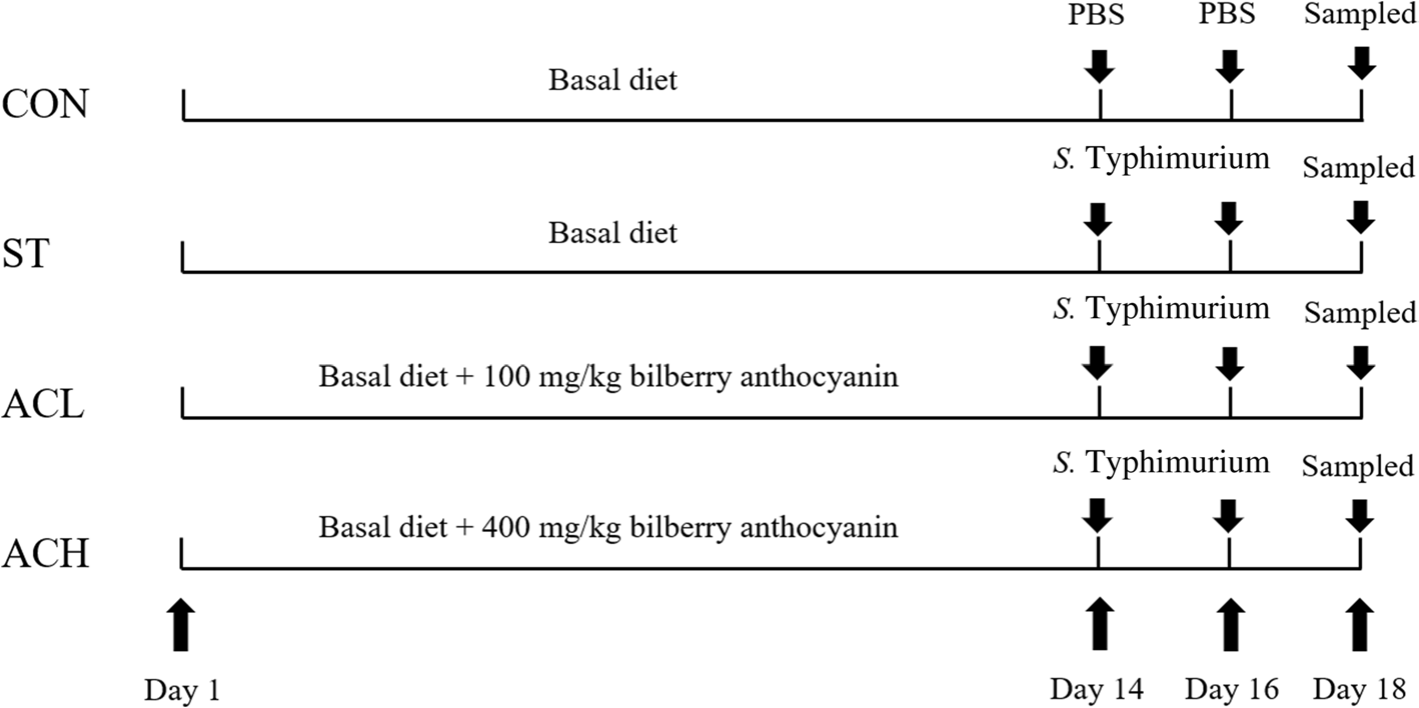
Schematic diagram of the experimental treatments

As shown in Fig. 1, birds in the control group (CON) and *Salmonella* infected group (ST) were fed the basal diets, and other birds in low dose of AC group (ACL) and high dose of AC group (ACH) were fed the basal diets supplemented with 100 or 400 mg/kg bilberry AC (Tianjin Jianfeng Natural Product R&D Co., Ltd., Tianjin, China; the purity > 36%), respectively. At d 14 and 16, birds in ST, ACL and ACH were orally challenged with 1.0 × 10^9^ colony forming units (CFUs) of *Salmonella *Typhimurium in phosphate buffer saline (PBS), and birds in the CON were given the same volume of sterile PBS.

Chickens were allowed ad libitum access to fresh water and mashed diets. The temperature of the room was maintained at approximately 34 °C during d 1 to 3, then decreased gradually by 3 °C each week to reach a constant temperature of 26 °C. Artificial light was provided continuously during d 1 to 3, then decreased gradually by 2 h each day to reach a constant illumination time of 16 h. Feed consumption was recorded every day and body weight (BW) was recorded on d 1, 18, 28 and 60. The average daily gain (ADG), average feed intake (ADFI), and feed to gain ratio (F/G) were calculated.

### Sample collection

At d 18, 2 birds closest to average BW of the replicate were deprived of feed for 12 h. Blood samples (5 mL) were taken from the wing vein and drawn into vacutainers with anticoagulant (BD™ Vacutainer, Franklin Lakes, NJ, USA), then the birds were euthanized. Plasma was obtained after centrifugation (3000 × *g*, 10 min) at room temperature. The liver and spleen were collected aseptically after the connective tissues were cleaned out. Ileal segments (around 1 cm long) were quickly fixed in 4% solution of paraformaldehyde. Furthermore, the samples of mucosa were gently scraped from the middle ileum, and cecal contents were collected. All samples were stored immediately at − 80 °C until analysis.

### Determination of *Salmonella* colonization

The colonization of *Salmonella* in liver and spleen were determined as previously described [16]. Samples were homogenized in sterile PBS containing 0.1% Triton X-100, then diluted to 10%, 1%, and 0.1%. Each sub-dilution (100 μL) was evenly spread on *Salmonella-Shigella* (SS) agar plates and cultured in a constant temperature incubator (37 °C) until bacteria were grown.

### Morphological analysis of ileum

Ileal segments were fixed with polyformaldehyde solution (4%) for more than 48 h, then dehydrated and processed into paraffin sections. Dewaxed sections were rehydrated, mounted, stained with hematoxylin-eosin (H&E), and then observed under the PANNORAMIC SCAN II (3DHISTECH, Budapest, Hungary). Villus height and crypt depth were measured on 5 fields for each section, and the ratio of villus height to crypt depth (V/C) was calculated.

### Determination of nitric oxide in plasma and inflammatory cytokines in ileal mucosa

Nitric oxide (NO) concentration in plasma was determined with a commercial kit (Nanjing Jiancheng Bioengineering Institute, Nanjing, China); the contents of interleukin 1β (IL-1β), IL-6, IL-8, tumor necrosis factor-α (TNF-α), interferon-β (IFN-β), and IFN-γ in ileal mucosa were determined with chicken ELISA kits (Jiangsu Meimian Industrial Co., Ltd., Zhangjiagang, China) according to the manufacturer’s instructions, respectively.

### Quantitative real-time PCR (qRT-PCR) analysis

The mRNA expression of zonula occludens 1 (*ZO*-1), occludin, claudin-1 and mucin 2 (*MUC*2) in ileal mucosa were determined. As previously described [17], total RNA was extracted from powdered frozen samples and reverse transcribed using RNAiso Plus and PrimeScript™II 1st Strand cDNA Synthesis Kits (Takara, Tokyo, Japan), respectively. Real-time PCR was performed on the CFX96 RT-PCR Detection System (Bio-Rad, CA, USA). Primers used are shown in Table 2. β-actin was used as a housekeeping gene, and 2^–△△Ct^ method was used to quantify relative mRNA expression levels of genes.

**Table 2:** Primer sequences for real-time PCR

Gene	GenBank ID	Primer sequence (5′ to 3′)
*ZO-1*	NM_040680628.1	F: CCAAAGACAGCAGGAGGAGA
		R: TGGCTAGTTTCTCTCGTGCA
Claudin-1	NM_001013611.2	F: GAGGATGACCAGGTCAAGAAG
		R: TGCCCAGCCAATGAAGAG
Occludin	NM_205128.1	F: TCATCCTGCTCTGCCTCATCT
		R: CATCCGCCACGTTCTTCAC
*MUC2*	NM_040673077.1	F: CATTCAACGAGGAGAGCTGC
		R: TTCCTTGCAGCAGGAACAAC
β-actin	NM_205518.1	F: GAGAAATTGTGCGTGACATCA
		R: CCTGAACCTCTCATTGCCA

*F* Forward primer, *R* Reverse primer, *ZO-1 *Zonula occludens 1, *MUC2 *Mucin 2

### Microbial DNA extraction and 16S rRNA sequencing

Microbial DNA was extracted from cecal contents (FastDNA® Spin Kit for Soil, MP Biomedical, Santa Ana, CA, USA) according to standard procedures. The DNA extract was checked and determined with NanoDrop 2000 UV-vis spectrophotometer (Thermo Scientific, Wilmington, DE, USA). The hypervariable region V3 to V4 of the bacterial 16S rRNA gene was amplified by using the 338F/806R primer pairs (338F: 5′-ACTCCTACGGGAGGCAGCAG-3′; 806R: 5′-GGACTACHVGGGTWTCTAAT-3′) and the sequencing was performed (Illumina NovaSeq PE250, San Diego, CA, USA). Raw sequences were quality-filtered and clustered into operational taxonomic units (OTUs) at 97% similarity by UPARSE (http://www.drive5.com/uparse) version 11.0.667, moreover, the chimeric sequences were identified and removed. Each OTU representative sequences were analyzed by RDP Classifier (https://sourceforge.net/projects/rdp-classifier) version 2.10, based on the release 138 the SILVA database (https://www.arb-silva.de).

### Statistical analysis

Statistics of growth performance, bacteria load, histological measurements, biochemical variables, and gene expression levels were examined by one-way analysis of variance (ANOVA) in SPSS 20.0 for Windows (SPSS, Chicago, IL, USA). When treatment effects were significant (*P* < 0.05). Duncan’s multiple range tests were used to compare the individual means. Tabulated results were shown as means with SEM derived from the ANOVA error mean square.

For data of cecal microbiota, Venn diagram, community barplot, and community heatmap were created with R software (http://www.R-project.org) version 3.3.1 statistics and mapping. Alpha-diversity (Chao1, Pd, Shannon, and Sobs indexes) were calculated using Mothur (https://www.mothur.org) version 1.30.2, and Welch’s *t*-test was used to compare post-hoc means. Beta-diversity was evaluated by principal co-ordinates analysis (PCoA) to show the similarity and differences of community structures among different samples, and ANOSIM was used to test the significance of separation via R software (http://www.R-project.org) version 3.3.1. The non-parametric Kruskal-Wallis sum-rank test was used to detect the differences of abundance in species among different groups and obtain significantly different species. Wilcoxon Rank-sum test was used to test the difference consistency of the different species in the previous step. Linear discriminant analysis (LDA) of effect size (LEfSe) was used to estimate the impact of these different species on the difference between groups. Using Phylogenetic Investigation of Communities by Reconstruction of Unobserved State (PICRUSt2) (https://github.com/picrust/picrust2) version 2.2.0, the corresponded the Kyoto Encyclopedia of Genes and Genomes (KEGG) Ortholog (KO) information through the OTUs greengene ID to obtain and the relative abundance of KO was calculated. The abundance of pathway levels 2 functional categories was calculated based on the KEGG database (http://www.genome.jp/kegg) and corresponding OTUs abundance. Relative abundance of microbial composition and function were analyzed and visualized using Statistical Analysis of Metagenomic Profiles (STAMP) (https://beikolab.cs.dal.ca/software/STAMP) version 2.1.3 with 95% confidence interval. Welch’s *t*-test was used to compare post-hoc means.

## Results

### Growth performance

As shown in Table 3, *Salmonella* infection decreased (*P* < 0.05) the BW of birds at d 18, 28 and 60. From d 1 to 18, d 1 to 28 and d 1 to 60, the ADG of birds in the infected birds (ST) were all decreased (*P* < 0.05), while the F/G increased (*P* < 0.05), i.e., less efficient compared with both variables in CON. In addition, the ADG from d 1 to 18 was increased and F/G was decreased (*P* < 0.05) in the ACL and ACH compared with those in the challenged but non-supplemented birds (ST). There was no significant (*P* > 0.05) difference in average daily feed intake among treatments.

**Table 3:** Effect of bilberry anthocyanin on growth performance in chickens challenged with *Salmonella*

Variable	Treatments				SEM	***P***-value
	CON	ST	ACL	ACH		
Body weight, g						
Day 1	28.00	28.05	28.03	28.05	0.01	0.268
Day 18	177.75^a^	164.08^b^	178.00^a^	182.50^a^	1.49	< 0.001
Day 28	366.40^a^	335.90^b^	352.20^ab^	351.25^ab^	3,37	0.026
Day 60	1227.11^a^	1167.34^b^	1189.90^ab^	1195.82^ab^	8.30	0.043
Average daily gain, g						
Day 1 to 18	8.32^a^	7.56^b^	8.33^a^	8.58^a^	0.08	< 0.001
Day 1 to 28	12.08^a^	11.07^b^	11.55^ab^	11.53^ab^	0.12	0.026
Day 1 to 60	20.50^a^	19.31^b^	19.60^ab^	19.81^ab^	0.16	0.030
Average daily feed intake, g						
Day 1 to 18	13.77	13.72	13.51	13.53	0.11	0.805
Day 1 to 28	24.51	24.20	24.29	24.12	0.35	0.343
Day 1 to 60	42.77	42.47	41.16	41.26	0.27	0.070
Feed to gain ratio						
Day 1 to 18	1.75^b^	1.93^a^	1.72^b^	1.66^b^	0.02	< 0.001
Day 1 to 28	2.02^c^	2.18^a^	2.09^b^	2.09^b^	0.01	0.010
Day 1 to 60	2.09^b^	2.19^a^	2.10^b^	2.10^b^	0.02	0.026

*CON* Control group, *ST Salmonella* infected group, *ACL* Low dose of anthocyanin group, *ACH* High dose of anthocyanin group, *SEM* Standard error of the mean

^a–c^Means within a row with different superscripts are significantly different (*P* < 0.05)

### Bacterial load in the liver and spleen

As shown in Table 4, *Salmonella* infection increased (*P* < 0.05) the number of *Salmonella* cells in the liver and spleen of chickens. Compared with the ST-infected birds, supplementation with both 100 mg/kg and 400 mg/kg of AC decreased about 10-fold (*P* < 0.05) the number of *Salmonella* cells in the liver and spleen of birds.

**Table 4:** Effect of bilberry anthocyanin on bacterial load in liver and spleen of 18-d chickens challenged with *Salmonella*

Item	Treatments				SEM	***P***-value
	CON	ST	ACL	ACH		
log_10_ CFU/g liver	0.00^c^	2.65^a^	1.36^b^	1.42^b^	0.26	< 0.001
log_10_ CFU/g spleen	0.00^c^	2.75^a^	1.80^b^	1.35^b^	0.25	< 0.001

*CON* Control group, *ST Salmonella* infected group, *ACL* Low dose of anthocyanin group, *ACH* High dose of anthocyanin group, *SEM* Standard error of the mean

^a-c^Means within a row with different superscripts are significantly different (*P* < 0.05)

### Ileal mucosal morphology

**Fig. 2:**
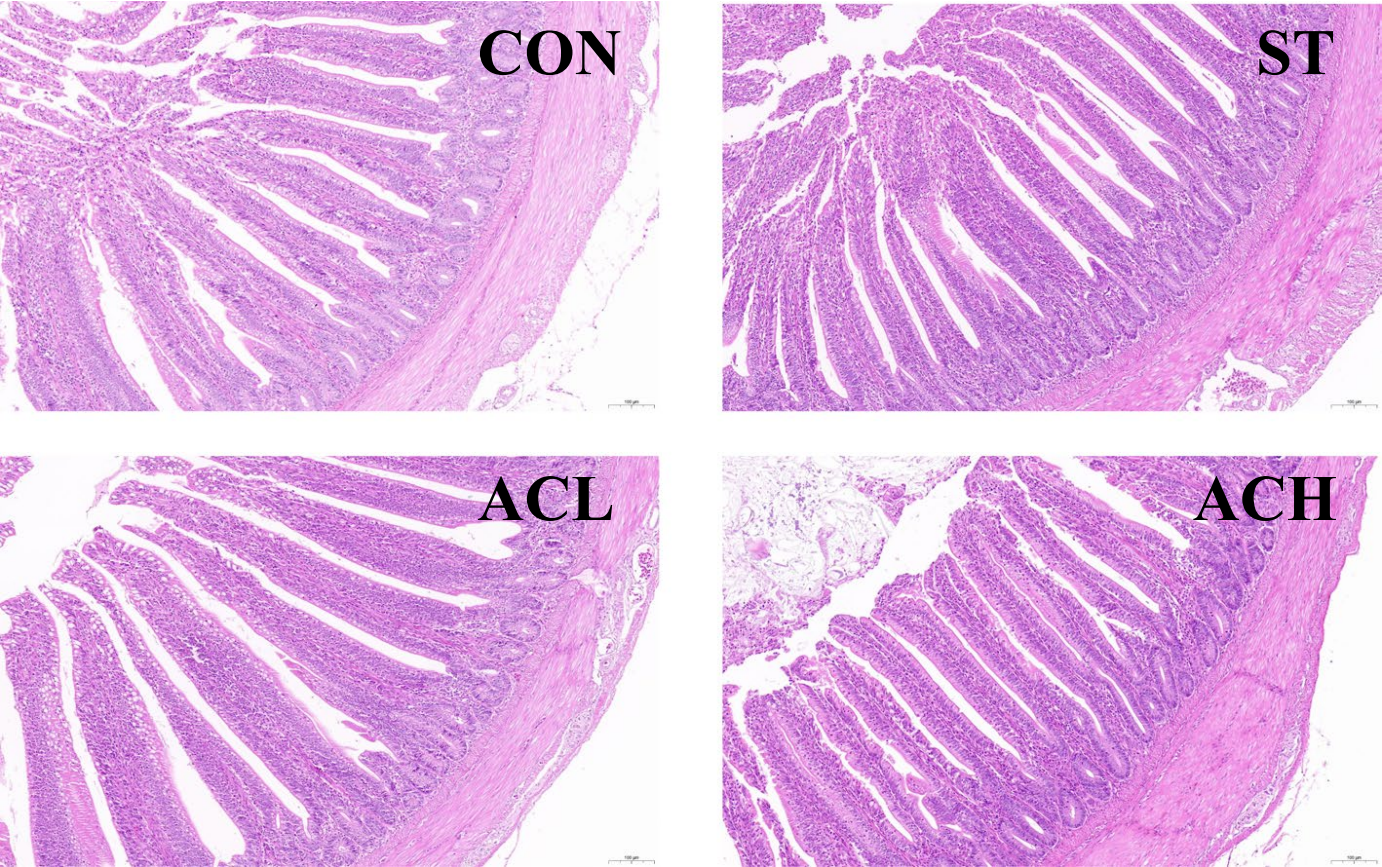
Hematoxylin-eosin (H&E) stained ileal mucosa. Scale bar at 100 μm. CON, control group; ST, *Salmonella* infected group; ACL, low dose of anthocyanin group; ACH, high dose of anthocyanin group

As shown in Table 5, compared with the non-infected controls, both the ileal villus height and V/C were decreased (*P* < 0.05) in the ST, and the ileal crypt depth was significantly increased. Compared with the infected birds (ST), supplementation with AC increased (*P* < 0.05) the ileal villus height, and 400 mg/kg of AC treatment reduced ileal crypt depth and increased V/C (*P* < 0.05). Representative images of H&E staining of ileal mucosa were shown in Fig. 2.

**Table 5:** Effect of bilberry anthocyanin on ileal morphology in 18-d chickens challenged with *Salmonella*

Variable	Treatments				SEM	***P***-value
	CON	ST	ACL	ACH		
Villus height, μm	0.64^a^	0.52^b^	0.71^a^	0.70^a^	0.02	0.012
Crypt depth, μm	0.14^b^	0.18^a^	0.17^ab^	0.15^b^	0.01	0.027
V/C	4.42^a^	3.14^b^	3.87^ab^	4.48^a^	0.17	0.015

*CON* Control group, *ST Salmonella* infected group, *ACL* Low dose of anthocyanin group, *ACH* High dose of anthocyanin group, *V/C* The ratio of villus height to crypt depth, *SEM* Standard error of the mean

^a,b^Means within a row with different superscripts are significantly different (*P* < 0.05)

### Biochemical variables

**Fig. 3:**
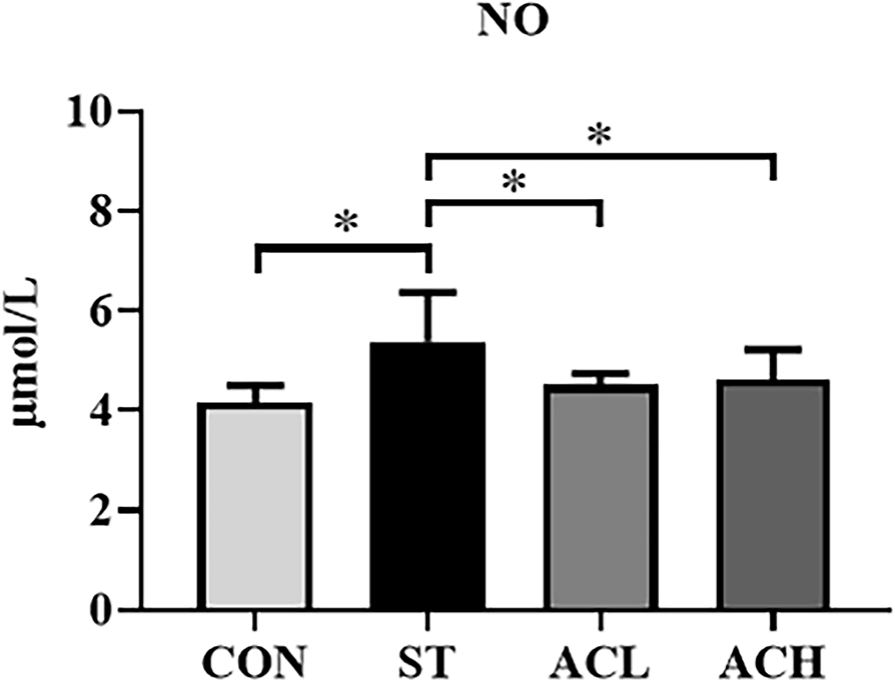
Effect of bilberry anthocyanin on plasma NO content in 18-d chickens challenged with *Salmonella.* CON, control group; ST, *Salmonella* infected group; ACL, low dose of anthocyanin group; ACH, high dose of anthocyanin group; NO, Nitric oxide. Date were shown as means ± SEM (*n* = 12), ^*^P < 0.05

As shown in Fig. 3, the plasma NO content of chickens in the ST treatment was higher (*P* < 0.05) than that of the controls. Compared with the *Salmonella*-infected birds (ST), supplementation with both 100 mg/kg and 400 mg/kg of AC decreased (*P* < 0.05) plasma NO content, almost down to the level in control chickens.

As shown in Table 6*, Salmonella* infection significantly increased the ileal contents of inflammatory cytokines (IL-1β, IL-6, IL-8, TNF-α, IFN-β and IFN-γ) compared with the CON. These variables were all reduced (*P* < 0.05) in infected birds which supplemented with AC. In addition, the 100 mg/kg of AC treatment significantly increased ileal IL-1β and IL-6 contents in the chickens challenged with *Salmonella*, compared with the 400 mg/kg of AC treatment.

**Table 6:** Effect of bilberry anthocyanin on inflammatory cytokines in 18-d chickens challenged with *Salmonella*

Item	Treatments				SEM	***P***-value
	CON	ST	ACL	ACH		
IL-1β, pg/mg pro	119.23^b^	147.51^a^	81.16^c^	109.23^b^	5.25	< 0.001
IL-6, pg/mg pro	6.49^b^	8.10^a^	5.17^c^	6.22^b^	0.24	< 0.001
IL-8, pg/mg pro	25.62^b^	32.36^a^	20.51^b^	25.14^b^	1.23	0.004
TNF-α, pg/mg pro	17.51^b^	23.31^a^	14.65^b^	16.71^b^	0.75	< 0.001
IFN-β, pg/mg pro	15.09^b^	18.89^a^	11.93^b^	15.52^b^	0.73	0.006
IFN-γ, pg/mg pro	22.04^b^	28.19^a^	20.32^b^	22.54^b^	0.88	0.006

*CON* Control group, *ST Salmonella* infected group, *ACL* Low dose of anthocyanin group, *ACH* High dose of anthocyanin group, *IL-1β* Interleukin 1β, *IL-6* Interleukin 6, *IL-8* Interleukin 8, *TNF-α* Tumor necrosis factor-α, *IFN-β* Interferon-beta, *IFN-γ* Interferon-γ, *SEM* Standard error of the mean

^a–c^Means within a row with different superscripts are significantly different (*P* < 0.05)

### Gene expression in ileal mucosa

**Fig. 4:**
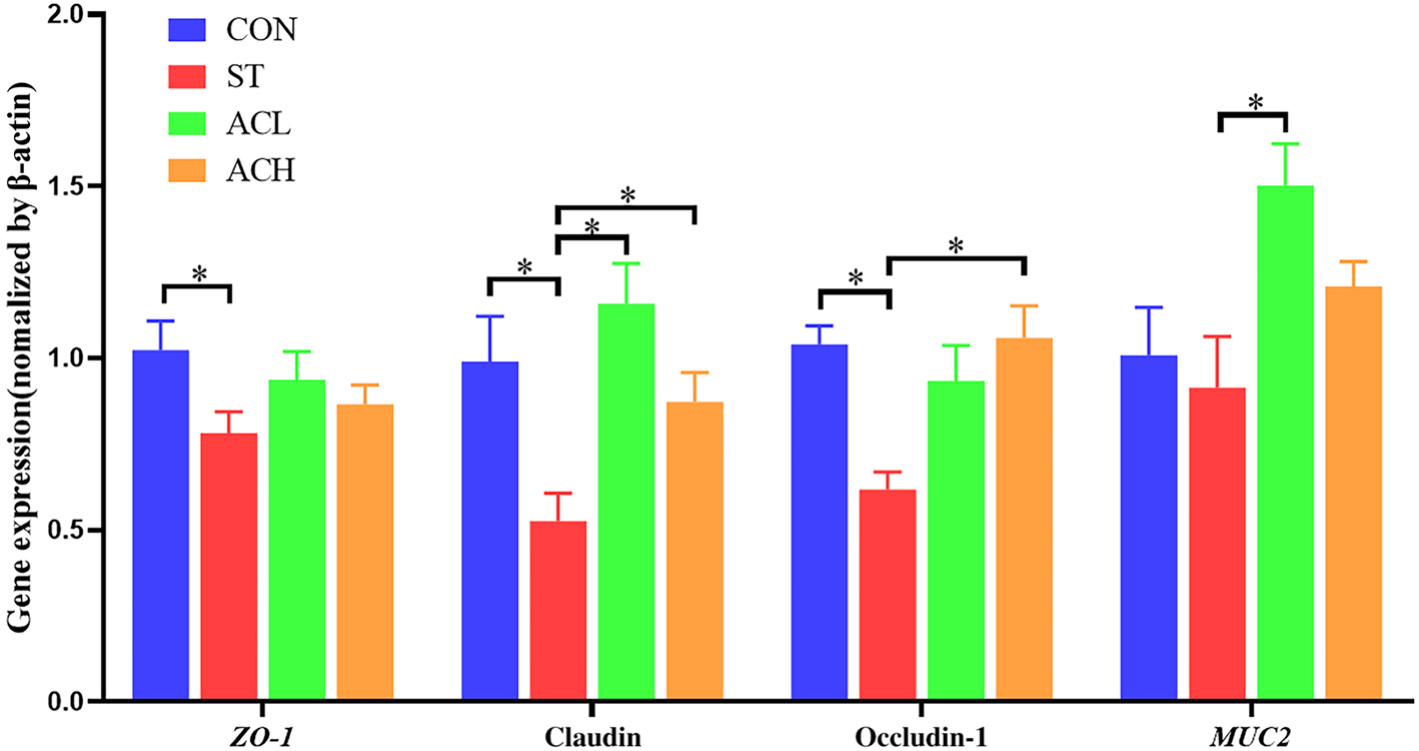
Effect of bilberry anthocyanin on gene expression in ileum. CON, control group; ST, *Salmonella* infected group; ACL, low dose of anthocyanin group; ACH, high dose of anthocyanin group; *ZO-1*, Zonula occluden 1; *MUC2*, Mucin 2. Date were shown as means ± SEM (*n* = 12), ^*^P < 0.05

The relative expression of barrier-related genes in ileal mucosa was shown in Fig. 4. *Salmonella* infection down-regulated (*P* < 0.05) the expression of *ZO-1*, claudin-1 and occludin, but had no effect on *MUC2* expression (*P* > 0.05). Compared with birds in ST, 100 mg/kg of AC up-regulated (*P* < 0.05) the expression of claudin-1 and *MUC2*, and 400 mg/kg of AC supplementation up-regulated (*P* < 0.05) the expression of *ZO-1* and occludin.

### Richness and diversity of cecal microbes

A total of 1,532,777 sequences (an average of 63,866 sequences per sample) were obtained, and the average length of the sequences was 413 bp. Overall, 697 OTUs were detected according to a nucleotide sequence identity of 97% between sequences.

**Fig. 5:**
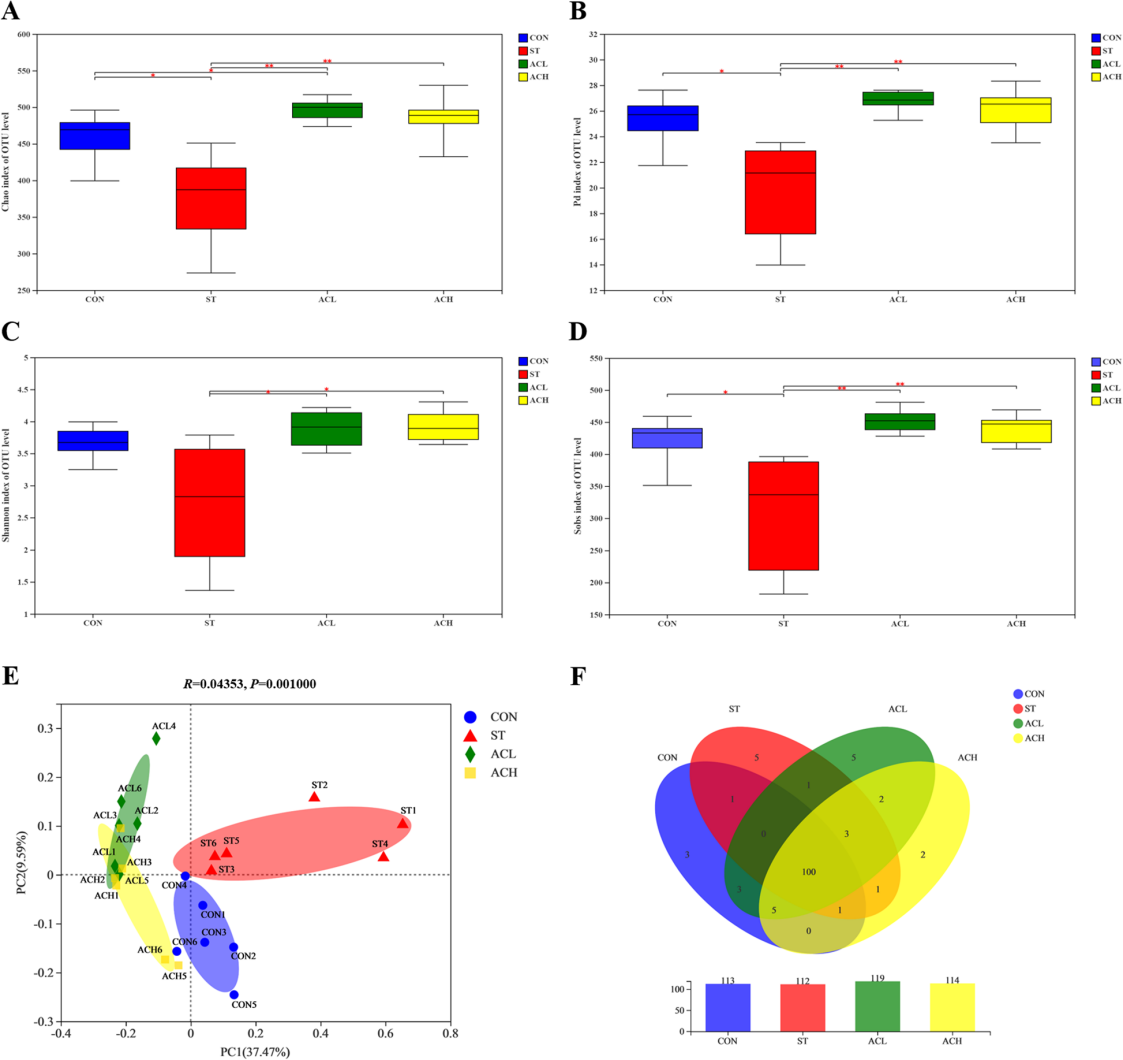
Effects of bilberry anthocyanin on the average richness and diversity of the cecal bacteria community. **A**–**D** The effects of AC on the Alpha-diversity (Chao1, Pd, Shannon, and Sobs indexes). **E** Principal co-ordinates analysis (PCoA) of bacterial communities in CON, ST, ACL, and ACH. **F** Venn diagram of bacterial communities in CON, ST, ACL, and ACH. CON, control group; ST, *Salmonella* infected group; ACL, low dose of anthocyanin group; ACH, high dose of anthocyanin group. Date were shown as mean ± SEM (*n* = 6), ^*^P < 0.05

Alpha-diversity of microbial communities were provided in Fig. 5A–D, *Salmonella* infection decreased (*P* < 0.05) Chao1, Pd, Shannon, and Sobs indexes compared with the CON. ACL and ACH treatments had higher (*P* < 0.05) Chao1, Pd, Shannon and Sobs indexes than the infected birds (ST). In addition, the PCoA chart (Fig. 5E) showed that the CON, ACL and ACH treatments, with some similarities between ACL and ACH, had some distinctive separation from the ST treatment.

The common and unique OTUs among the treatments at the genus level was shown in the Venn diagram (Fig. 5F). One hundred thirty-two OTUs were determined, and there were 100 common OTUs among the four groups. In addition, 3, 5, 5 and 2 OTUs were unique in the CON, ST, ACL and ACH, respectively.

### Composition of cecal microbes

**Fig. 6:**
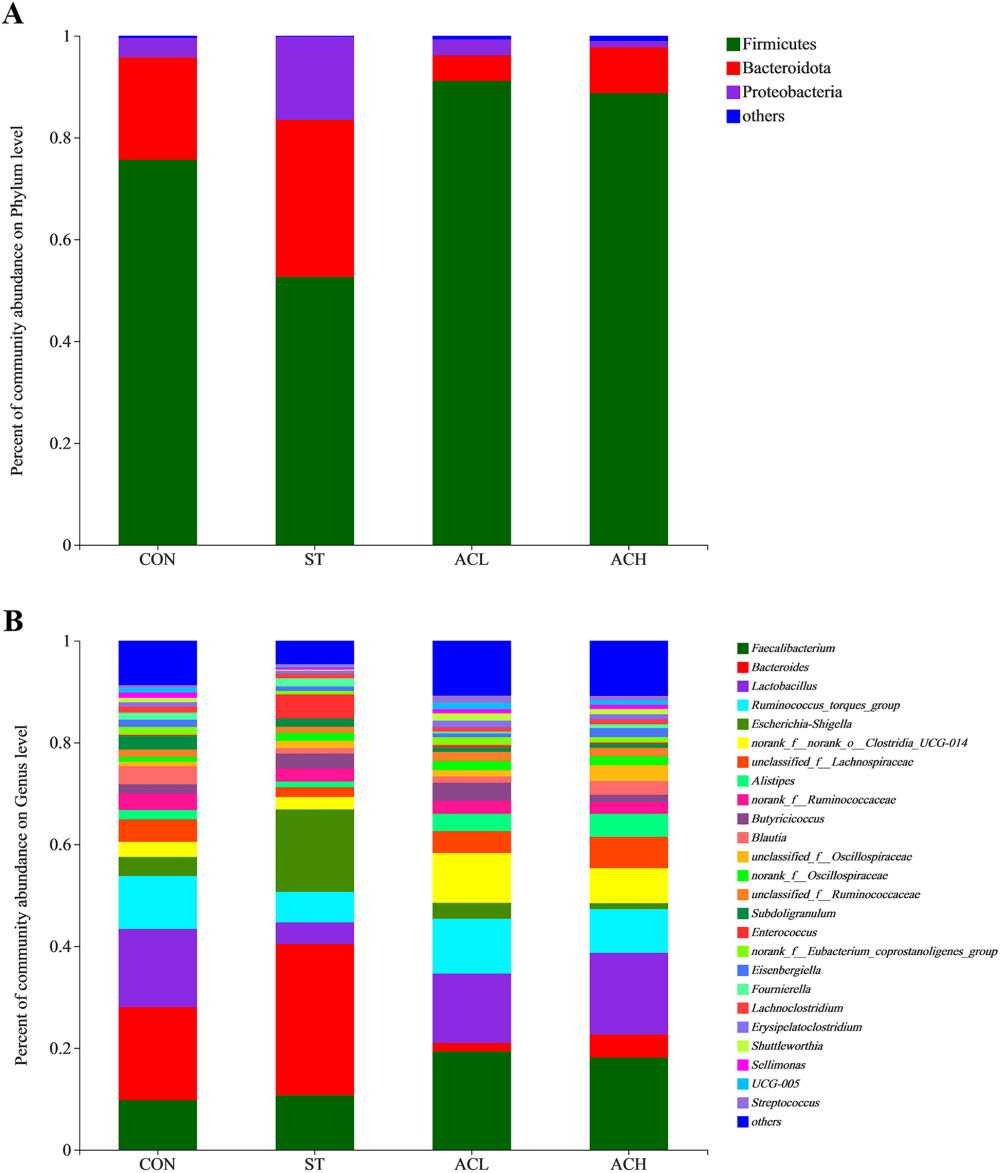
Relative abundance of bacterial composition in cecal contents at phylum (**A**) and genus (**B**) levels. The abscissa shows the name of the experimental group, the ordinate shows the proportion of the bacteria. Each color represents one bacterium, and the heigth of the band represents its percentage. CON, control group; ST, *Salmonella* infected group; ACL, low dose of anthocyanin group; ACH, high dose of anthocyanin group

**Fig. 7:**
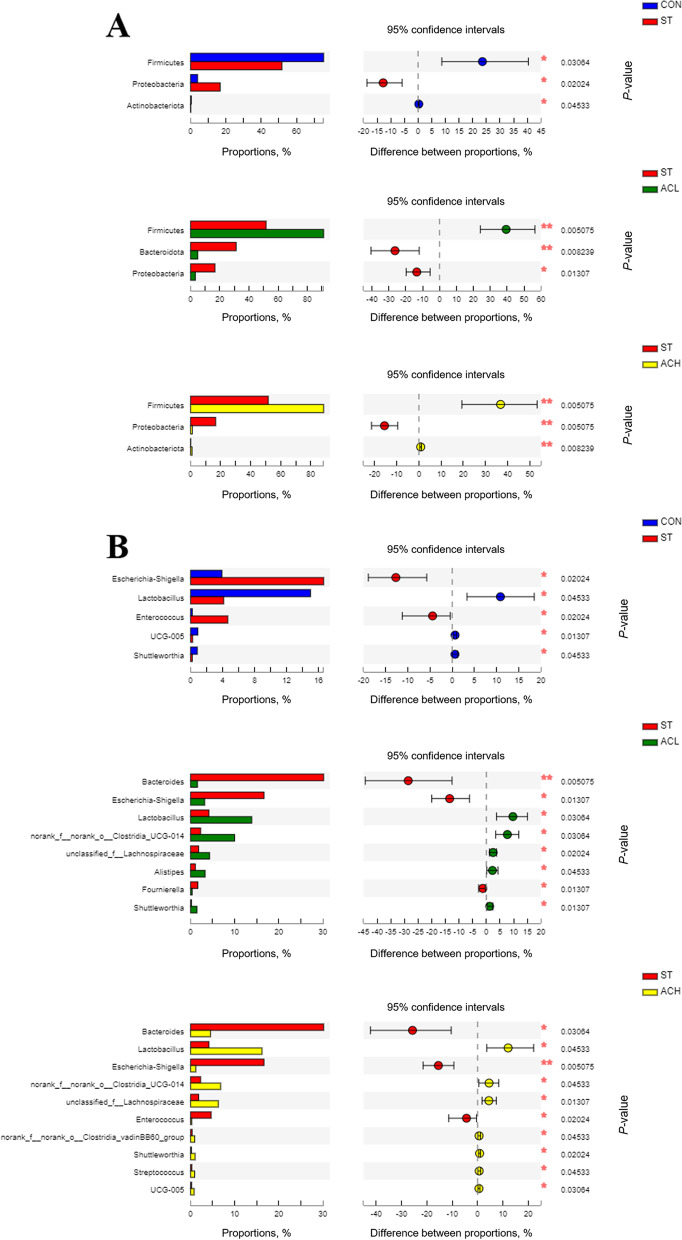
Differences in cecal microbes among treatments at the phylum (**A**) and genus (**B**) levels. Represent CON vs. ST, ST vs. ACL, and ST vs. ACH respectively. CON, control group; ST, *Salmonella* infected group; ACL, low dose of anthocyanin group; ACH, high dose of anthocyanin group

The relative abundance of bacteria at the phylum and genus level were presented in Figs. 6 and 7. Firmicutes, Bacteroidota and Proteobacteria were the top 3 phyla (> 1% at least in 1 of the 4 groups). From Fig. 7A, *Salmonella* infection decreased (*P* < 0.05) the relative abundance of Firmicutes, but increased (*P* < 0.05) the relative abundance of Proteobacteria compared with the CON. In comparison with the infected birds (ST), supplementation with AC (ACL and ACH) increased (*P* < 0.05) relative abundance of Firmicutes and decreased (*P* < 0.05) relative abundance of Proteobacteria, moreover, Bacteroidota was decreased (*P* < 0.05) in ACL and barely changed in ACH. As shown in Fig. 6B, *Faecalibacterium*, *Bacteroides*, *Lactobacillus*, *Ruminococcus* and *Escherichia-Shigella* were the major bacterial genera in cecal contents. Compared with the CON, *Salmonella* infection increased (*P* < 0.05) the relative abundances of *Escherichia-Shigella* and *Enterococcus*, but decreased (*P* < 0.05) the relative abundances of *UCG-005*, *Shuttleworthia* and *Lactobacillus*. Moreover, compared with birds in ST, in the ACL treatment the relative abundances of *Bacteroides*, *Escherichia-Shigella* and *Fournierella* were decreased (*P* < 0.05), along with increased (*P* < 0.05) *Lactobacillus*, *Clostridia_UCG-014*, *Lachnospiraceae*, *Alistipes* and *Shuttleworthia* (Fig. 7B); In the higher dose (ACH), the relative abundances of *Bacteroides*, *Escherichia-Shigella* and *Enterococcus* were decreased (*P* < 0.05), but those of *Lactobacillus*, *Clostridia_UCG-014*, *Lachnospiraceae*, *Clostridia_vadinBB60_group*, *Shuttleworthia*, *Streptococcus* and *UCG-005* were increased (*P* < 0.05).

**Fig. 8:**
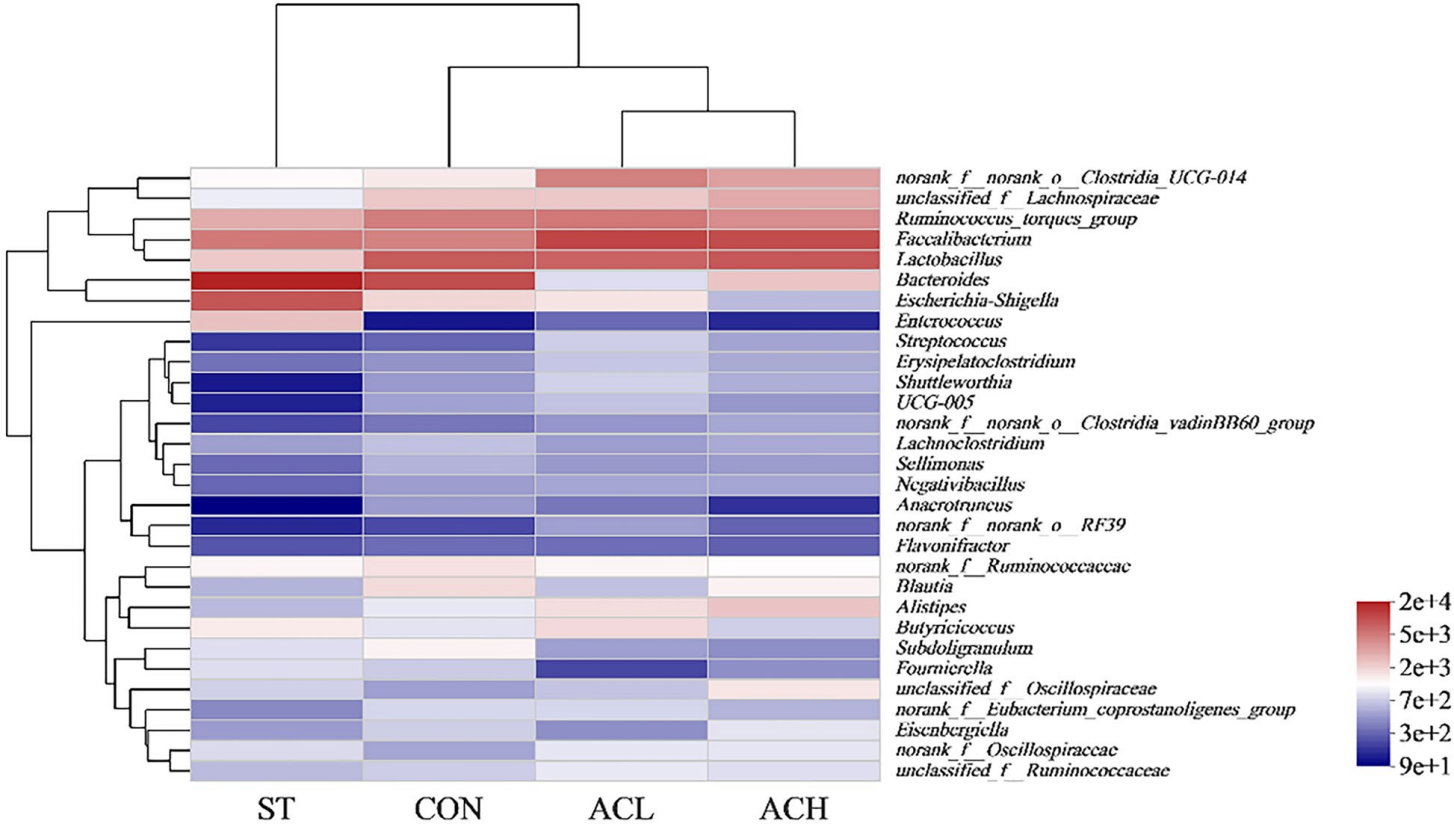
Clustering heatmap of bacterial community composition data at the genus level. The color blocks in the Heatmap represent the relative abundance of a genus, and the values were taken as the average values of each group of samples. Gradient from red to blue indicates high to low relative abundance. CON, control group; ST, *Salmonella* infected group; ACL, low dose of anthocyanin group; ACH, high dose of anthocyanin group

The 30 most abundant genera were clustered and analyzed by heatmap and cluster tree (Fig. 8). The results showed that the dominant bacterial communities in ACL, ACH and CON treatment groups were clustered together, and those in infected birds were clearly segregated from the other groups.

**Fig. 9:**
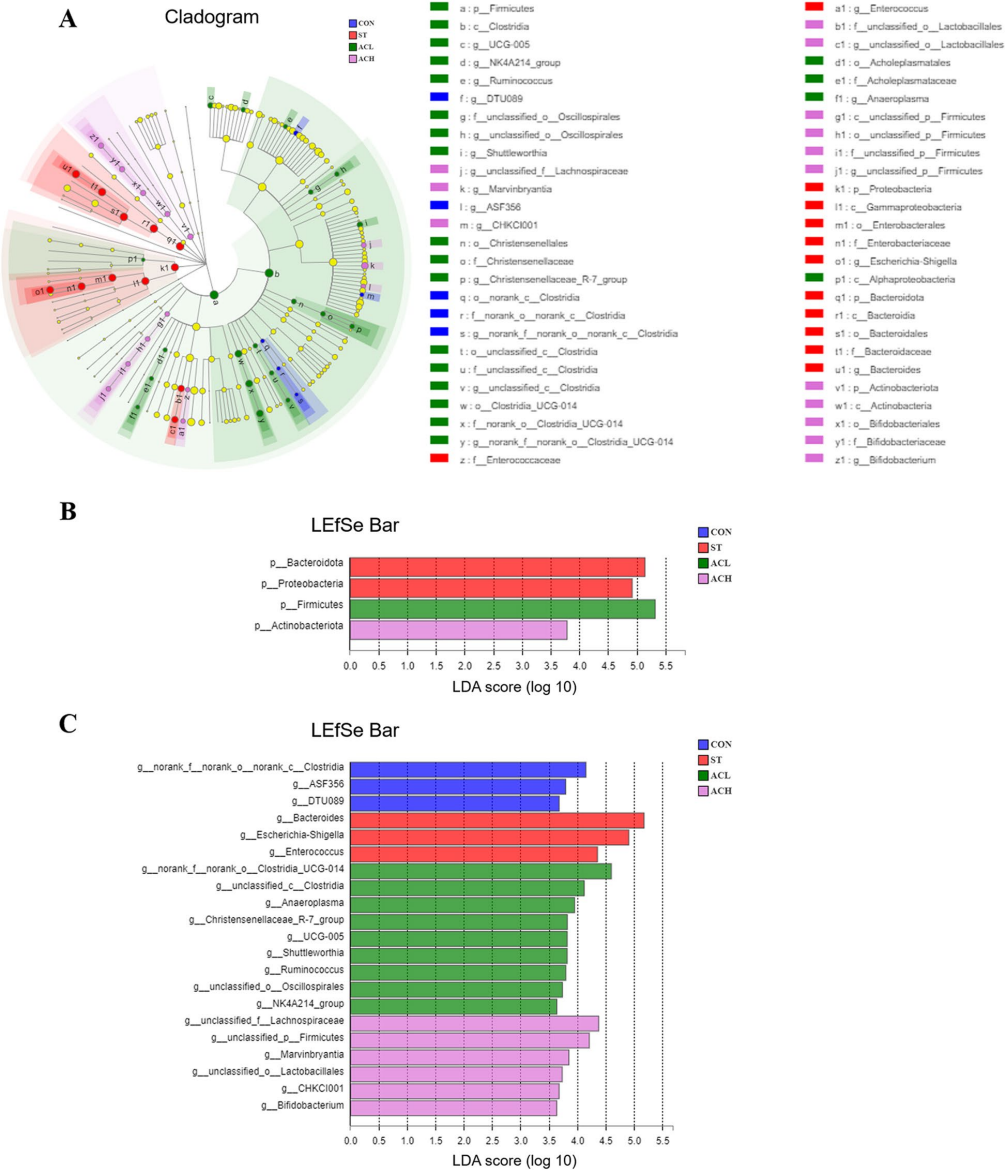
Different taxa microbe analysis in cecal contents based on LEfSe method. **A** Cladogram of the microbe structure axis. The circles from inside to out represent the classification level (phylum, class, order, family, and genus). The color of circles with letters mean that the bacteria was higher at CON, ST, ACL or ACH respectively, the diameter of each circle is proportional to the abundance of the group. LEfSe bar at phylum (**B**) and genus (**C**) level. The default parameters were LDA score > 2 and *P* < 0.05. Different-colored regions represent different constituents (blue: CON, red: ST, green: ACL, pink: ACH). CON, control group; ST, *Salmonella* infected group; ACL, low dose of anthocyanin group; ACH, high dose of anthocyanin group

As presented in the cladogram of the microbe structure axis (Fig. 9A), a significant change of microbes was found in CON, ST, ACL and ACH. The results of LEfSe confirmed the higher abundance of Bacteroidota and Proteobacteria in the ST, Firmicutes in the ACL, and Actinobacteriota in the ACH (Fig. 9B). In the ST, 3 genera were significantly more abundant, namely *Bacteroides*, *Escherichia-Shigella*, and *Enterococcus* (Fig. 9C). In addition, *Clostridia*, *ASF356*, and *DTU089* were significantly more abundant in the CON (Fig. 9C). Similarly, there were significant enrichment of *Clostridia_UCG-014*, *Clostridia*, *Anaeroplasma*, *Christensenellaceae_R-7_group*, *UCG-005*, *Shuttleworthia*, *Ruminococcus*, *Oscillospirales* and *NK4A213_group* in the ACL, and a profusion of *Lachnaspiraceae*, *Fimicutes*, *Marvinbryantia*, *Lactobacillales*, *CHKCI001* and *Bifidobacterium* in the ACH (Fig. 9C).

### Predicted function of cecal microbes

**Fig. 10:**
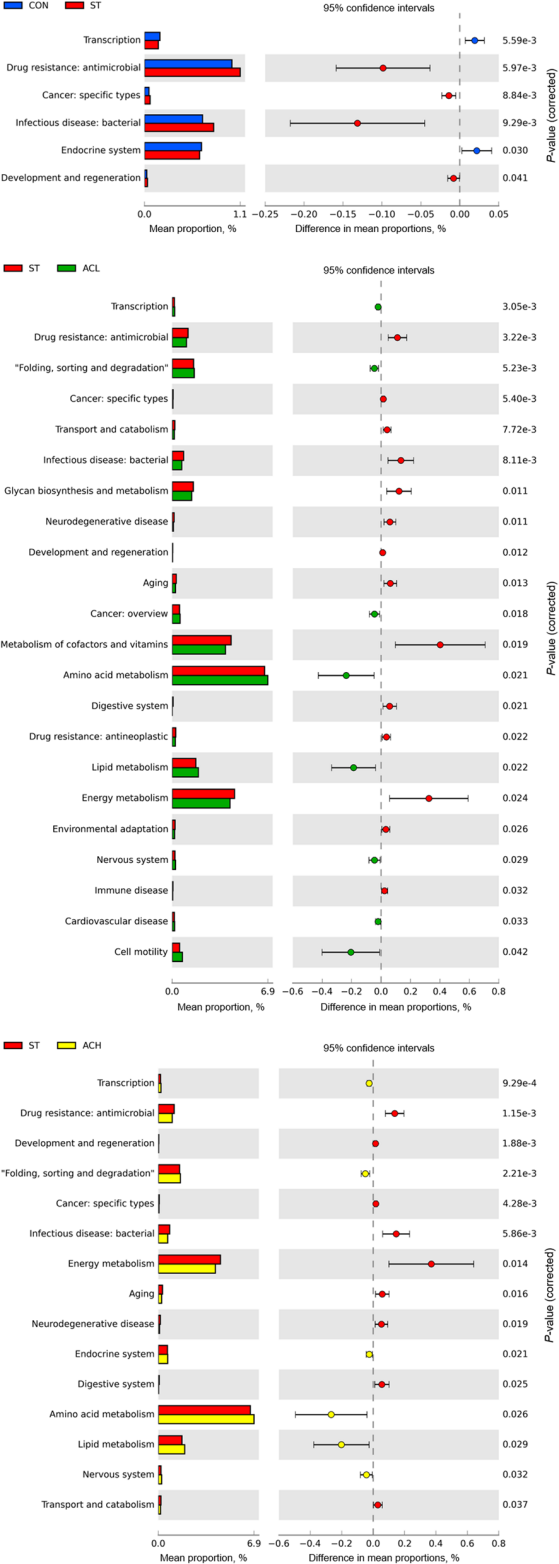
The relative abundance of KEGG pathway levels 2 functional analysis. Represent CON vs. ST, ST vs. ACL, and ST vs. ACH respectively. CON, control group; ST, *Salmonella* infected group; ACL, low dose of anthocyanin group; ACH, high dose of anthocyanin group

PICRUSt2 metagenome prediction (Fig. 10) showed that *Salmonella* infection enriched (*P* < 0.05) 4 pathways (drug antimicrobial resistance, specific cancer types, infectious bacterial disease, and development and regeneration), whereas it decreased (*P* < 0.05) transcription and endocrine system. Compared with the ST, dietary supplementation with AC (ACL and ACH) increased (*P* < 0.05) the abundances of 5 pathways, including transcription, ‘folding, sorting and degradation’, amino acid metabolism, lipid metabolism, cell motility and nervous system, while decreased (*P* < 0.05) the abundances of 9 pathways, including drug antimicrobial resistance, development and regeneration, specific cancer types, infectious bacterial disease, energy metabolism, aging, neurodegenerative disease, digestive system, and transport and catabolism.

## Discussion

### Growth performance

*Salmonella* infection causes diarrhea, damaging the immune system and composition of cecal microbes, resulting in seriously limited growth performance of chickens [18]. The present study confirmed *Salmonella* as a pathogen causing a decrease of growth performance in yellow-feathered chicken. Furthermore, AC alleviated weight loss and the increased F/G caused by *Salmonella* infection. However, there was no significant difference between 100 mg/kg and 400 mg/kg of AC in improving performance. A previous study showed that supplementation with cranberry AC did not improve growth performance of broilers [19], which was consistent with the research of bilberry AC on chickens [14]. These results indicated that AC did not directly promote growth of chickens and the main effect of AC on improving growth performance in the present study might be indirectly related to alleviating *Salmonella* infection, which also indicated the potentiality of AC as an antibiotic substitute for prevention of pathogens in poultry industry.

### Bacterial load in liver and spleen

Liver and spleen are the main immune organs of birds impacted by *Salmonella* infection [20], and *Salmonella* transferred to liver and spleen after colonizing the intestine, resulting in persistent infection in chickens [21, 22]. The present study found that after *Salmonella* infection, the number of *Salmonella* cells in the liver and spleen increased, but less so in birds fed diets supplemented with AC. This is in line with the reported anti-bacterial activities of AC, such as preventing *Salmonella enterititis* and *Escherichia coli*, which might be related to the ability to target and destroy lipopolysaccharide (LPS) in the cell wall, reducing adhesion and increasing the outflow of ATP from the cytoplasm [10, 23]. On the other hand, *Salmonella-*induced damages the intestinal barrier resulted in transfer of bacteria from gut to the organs via the bloodstream. These results indicated that AC ameliorated the extent of injury of the intestinal barrier.

### Intestinal barrier function

Intestinal barrier function, inflammatory cytokines levels, and composition of microbes together determine the intestinal health. It was worth noting that in some pathogen-infected models, antibiotics not only killed pathogenic bacteria but also affected the structure and diversity of normal microbes, thus had adverse influence on intestinal health [24]. Furthermore, previous studies in mice showed that antibiotic treatment resulted reduction in claudin 4 expression [25], destroyed the integrity of colon, triggered the transfer of pathogens to organs [26], and induced acute inflammation in the cecal mucosa [4]. Therefore, nutritional strategies of antibiotic substitute to improve intestinal health are needed.

The integrity of the intestinal barrier reflects conditions of the health and function of the intestine, and its damage is associated with decreased nutrient digestion and absorption, intestinal inflammation, as well as the transfer of pathogenic microbes and their metabolites such as endotoxin [27]. *Salmonella* infection causes the rupture and shedding of intestinal villi in chickens. Through the increasing of ileal villus height and V/C and the decreasing of ileal crypt depth, AC efficiently alleviated the ileal mucosal structure damages caused by *Salmonella*.

ZOs, claudins, occludin and MUC2 are the key components involved in intestinal barrier protection and immune response [28]. Previous studies showed that *Salmonella* infection induced intestinal barrier injury by suppressing gene expression of tight junction proteins (*ZO*-1, claudin-1 and occludin) and *MUC*2 [29, 30]. In the current experiment, dietary AC alleviated the adverse effects of chickens caused by *Salmonella* infection by up-regulating expression of *ZO*-1, claudin-1, occludin, and *MUC*2. Similarly, a previous study showed that substances containing AC reduced the impact of intestinal inflammatory damage by up-regulating the expression of *ZO*-1, claudin*-*1, occludin and *MUC*2 in high-fat diet-induced mice [31]. Based on the above results, AC clearly improved the integrity of the intestinal barrier, thereby alleviating intestinal damage in *Salmonella* infected chickens.

### Intestinal inflammation

Inflammatory responses are self-regulated processes recognizing and eliminating the invading pathogens and restoring the normal physiological function of tissue. Recent studies found that *Salmonella* infection induced large amounts of NO in macrophages of chickens [32], which promoted inflammatory responses in tissues and activated host immune function [33]. Then, NO reacted with O^2−^ to produce peroxynitrite, leading to lipid peroxidation of cell membranes and damage to DNA. A previous study found that AC reduced LPS-induced NO production in RAW264.7 cells [34], which was consistent with decreasing in plasma NO in current finding, indicating that AC alleviated *Salmonella*-induced immune responses.

The activation of intestinal mucosal immune function is mainly manifested by the releasing of cytokines. IL-1β is released rapidly in response to bacterial and viral infections and helps stimulate early innate immune responses. IL-6 acts as both proinflammatory and anti-inflammatory, responding quickly to *Salmonella* invasion [35]. IL-8 induces changes in cell morphology and enhances the antibacterial effect of immune cells. IFN-β affects the proliferation of immune cells and regulates the immune response, providing resistance to *Salmonella* infection [36]. IFN-γ and TNF-α were essential for the clearance of *Salmonella* in vivo [37, 38].

A previous study showed that *Salmonella* infection increased contents of IL-1β, IL-8, IFN-β, and IFN-γ in plasma of chickens [39]. Although the immune response plays an important role in eliminating pathogens, excessive release of inflammatory cytokines causes the destruction of tissue structure [40]. AC showed anti-inflammatory activity in vitro and in vivo, such as reducing the excessive release of pro-inflammatory mediator NO and cytokines (IL-1β, IL-6, IL-8, TNF-α etc.) in RAW 264.7 macrophages, and its intake was a strategy to prevent and inhibit inflammation [41, 42]. Also, bilberry extract inhibited intestinal inflammation in mice, reduced intestinal bleeding and improved intestinal histological structure [43]. In the current experiment, AC at a level of 100 mg/kg in the diet decreased the ileal cytokines (IL-1β, IL-6, IL-8, TNF-α, IFN-β, and IFN-γ) production in chickens challenged with *Salmonella*.

These results indicated that AC played a significant anti-inflammatory role by inhibiting the release of proinflammatory mediator and cytokines to reduce the inflammatory damage in the ileum, thereby limiting the infection of *Salmonella*.

### Diversity of intestinal microbiota

*Salmonella* infection inhibited the colonization of other normal microbiota in the ileum of chickens [44] and affected the composition and diversity of the ileal and cecal microbiota [32]. In the present research, *Salmonella* infection caused a decrease in cecal microbial diversity. Dietary AC increased alpha-diversity including Chao1, Pd, Shannon, and Sobs indexes, which might contribute to the richer and more stable intestinal microbes. Using β-diversity to indicate the degree of similarity between microbial communities, dietary supplementation with AC affected the structure of cecal microbes, making it different from that in *Salmonella*-infected chickens. The author inferred that the increase of diversity might be correlated with improving the integrity of intestinal barrier and the development of normal microbiota in *Salmonella*-infected chickens by dietary AC. Moreover, abundant hindgut microbes were beneficial to the ability to degrade AC that were not absorbed by the upper digestive tract, and the metabolites produced would be further utilized by intestinal epithelial cells [45].

### Composition of intestinal microbiota

Firmicutes, Bacteroidetes and Proteobacteria were the major phyla of all detected in chickens [46]. *Salmonella* infection resulted in the increasing of pathogenic and facultative anaerobic bacteria in the cecal microbiota of chickens, which destabilized the microbiota [47]. Supplementation with AC were effective in regulating the composition of cecal microbes in mice [48, 49]. In the present experiment, Firmicutes was increased and Proteobacteria was decreased by supplementation with AC. Proteobacteria contains various of pathogens such as *Salmonella*, *Escherichia coli*, and *Shigella*. At the genus level, *Escherichia-shigella* in Proteobacteria and *Enterococcus* were dominant in infected chickens; these were potentially harmful bacteria, triggering inflammation through up-regulating *Hsps* and inflammation genes [50, 51]. In addition, *Escherichia-Shigella* was correlated negatively with growth in chickens [29], and it activated NLRP3 inflammasomes and induced inflammation in broilers, associating with up-regulating *IL-6* expression [52, 53]. In the current experiment, AC supplementation decreased the abundance of these pathogens, thus alleviated of intestinal disease. Also, AC increased the abundance of beneficial bacteria, such as *Bacteroidetes*, *Lactobacillus, Clostridium* etc. *Bacteroidetes* was participated in the metabolism of feeding, maintained the stability of intestinal environment [54, 55], and inhibited the growth of pathogenic bacteria by producing antimicrobial peptides [56]. *Lactobacillus* inhibited the proliferation of other bacterial species by synthesizing biotin and producing lactic acid through fermentation, which thereby reducing intestinal pH and inhibiting the activity of pathogens [57, 58]. In addition, *Bacteroides*, *Clostridium*, *Ruminococcus*, *Alistipes* and *Lactobacillus* hydrolyzed starch and other macromolecules to form short-chain fatty acids through fermentation; these end-products showed anti-inflammatory activity when absorbed [46]. The increase of these beneficial bacteria thereby alleviating intestinal inflammation caused by *Salmonella* infection.

### Functional prediction of intestinal microbiota

Using PICRUSt2 to predict the potential function of microbial communities, *Salmonella* infection significantly enriched diseased-related pathways, such as drug antimicrobial resistance and infectious bacterial disease. Virulence genes and antibiotic resistance enhanced virulence of bacteria [3] and brought benefits to *Salmonella* survival in adverse environments [59]. The reduction of those enriched pathways in dietary AC group indicated that AC might inhibit the intestinal colonization and transfer of *Salmonella*-infected chickens by reducing the expression of *Salmonella* virulence and drug resistance genes.

Moreover, genomic prediction showed that, dietary AC significantly increased the enrichment of amino acid metabolism, lipid metabolism, and decreased the enrichment of transport and catabolism, and energy metabolism in *Salmonella*-infected chickens. The author inferred that the changes in these pathways might relate to the reduction of weight loss in *Salmonella*-infected chickens by dietary AC. After *Salmonella* invading the intestine, the intestinal mucosal immune system of host was activated through recognition of pathogen-associated molecular patterns such as flagellin and LPS [60]. In the present experiment, 100 mg/kg of AC decreased the enrichment of immune disease in *Salmonella*-infected chickens, which might contain some connection between reduction in immune system activation and secretion of immune cytokines caused by infection. Overall, dietary AC offset the negative effects caused by *Salmonella* infection through comprehensive regulation on microbiota composition and function.

## Conclusion

*Salmonella *Typhimurium infection of starter-phase chickens reduced weight gain, caused severe ileal inflammatory injury, and destroyed the optimal composition of cecal microbes. Dietary AC reduced *Salmonella* colonization in liver and spleen of challenged chickens, decreased secretion of inflammatory cytokines, up-regulated the expression of ileal genes encoding mucosal tight junction and mucin proteins, and modulated the composition of cecal microbes. In the current study, there was no significant difference between 100 and 400 mg/kg of AC in the parameters measured of *Salmonella*-infected chickens, therefore 100 mg/kg of AC was recommended to diets in broiler production to combat *Salmonella* infection.

## Data Availability

All data generated or analyzed during this study are available from the corresponding author on request.

## Abbreviations

AC: Anthocyanins
ACH: High dose of anthocyanin group
ACL: Low dose of anthocyanin group
ADG: Average daily gain
ANOVA: Analysis of variance
BW: Body weight
CFU: Colony forming unit
CON: Control group
F/G: Feed to gain ratio
H&E: Hematoxylin-eosin
IFN-β: Interferon-β
IFN-γ: Interferon-γ
IL-1β: Interleukin 1β
IL-6: Interleukin 6
IL-8: Interleukin 8
KEGG: Kyoto Encyclopedia of Genes and Genomes
KO: KEGG Ortholog
LDA: Linear discriminant analysis
LESfe: Linear discriminant analysis of effect size
LPS: Lipopolysaccharide
MUC2: Mucin 2
NO: Nitric oxide
OTUs: Operational taxonomic units
PBS: Phosphate buffer saline
PCoA: Principal co-ordinates analysis
PICRUSt2: Phylogenetic Investigation of Communities by Reconstruction of Unobserved States
qRT-PCR: Quantitative real-time PCR
SEM: Standard error of the mean
ST: *Salmonella* infected group
STAMP: Statistical Analysis of Metagenomic Profiles
TNF-α: Tumor necrosis factor-α
V/C: The ratio of villus height to crypt depth
ZO-1: Zonula occluden 1
